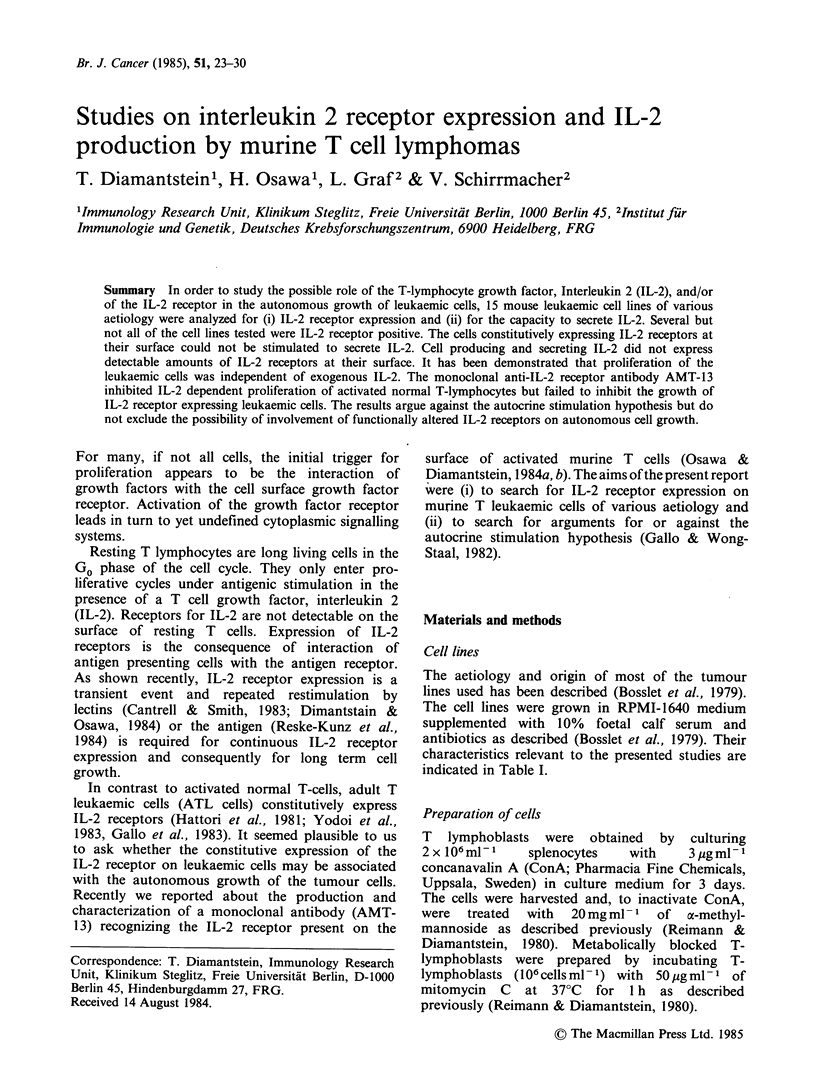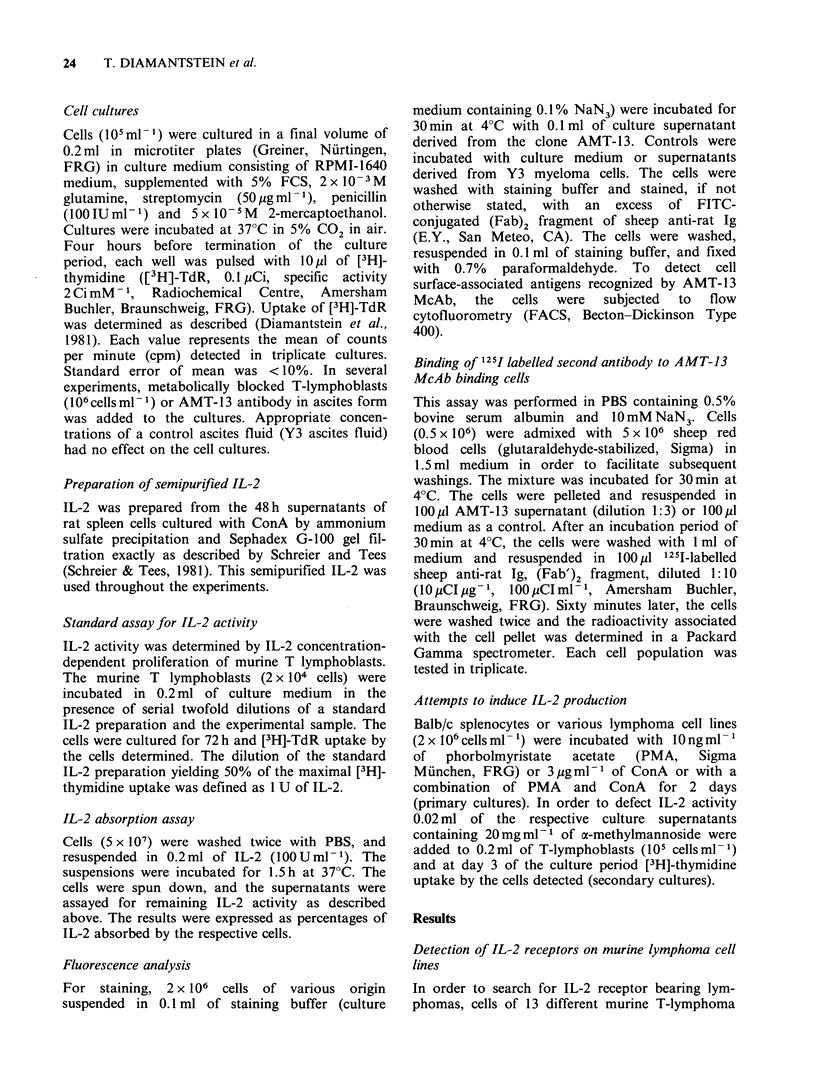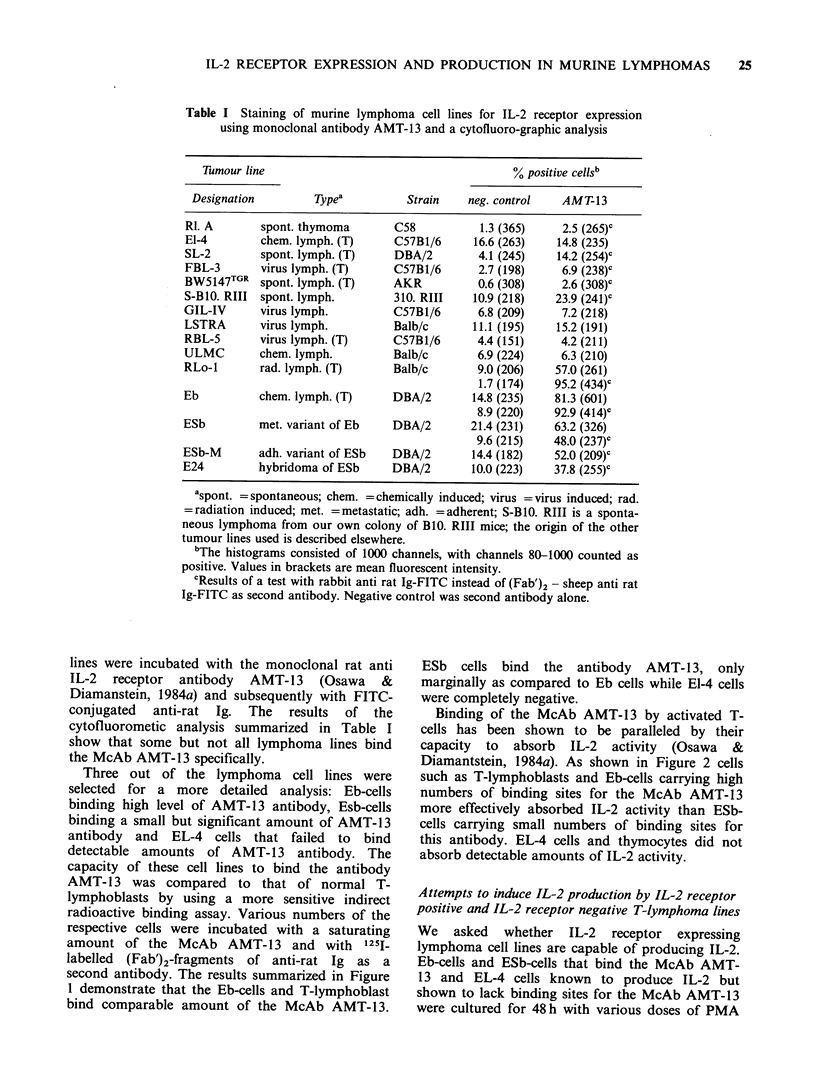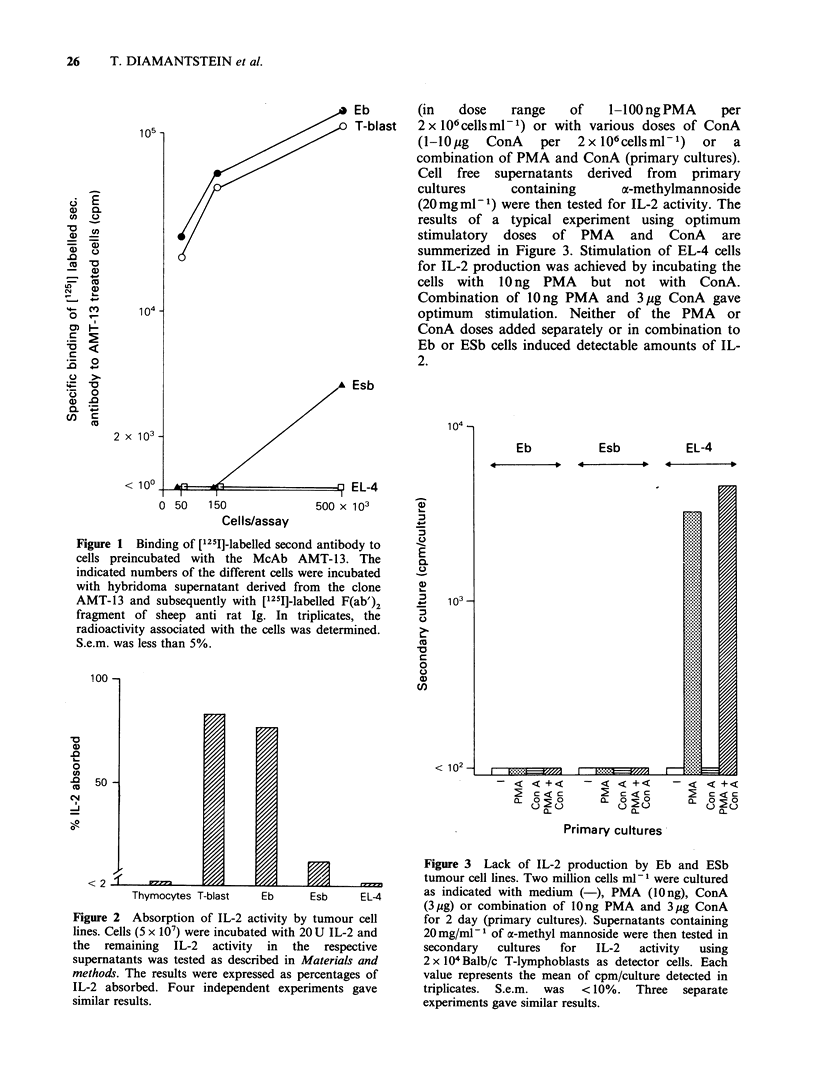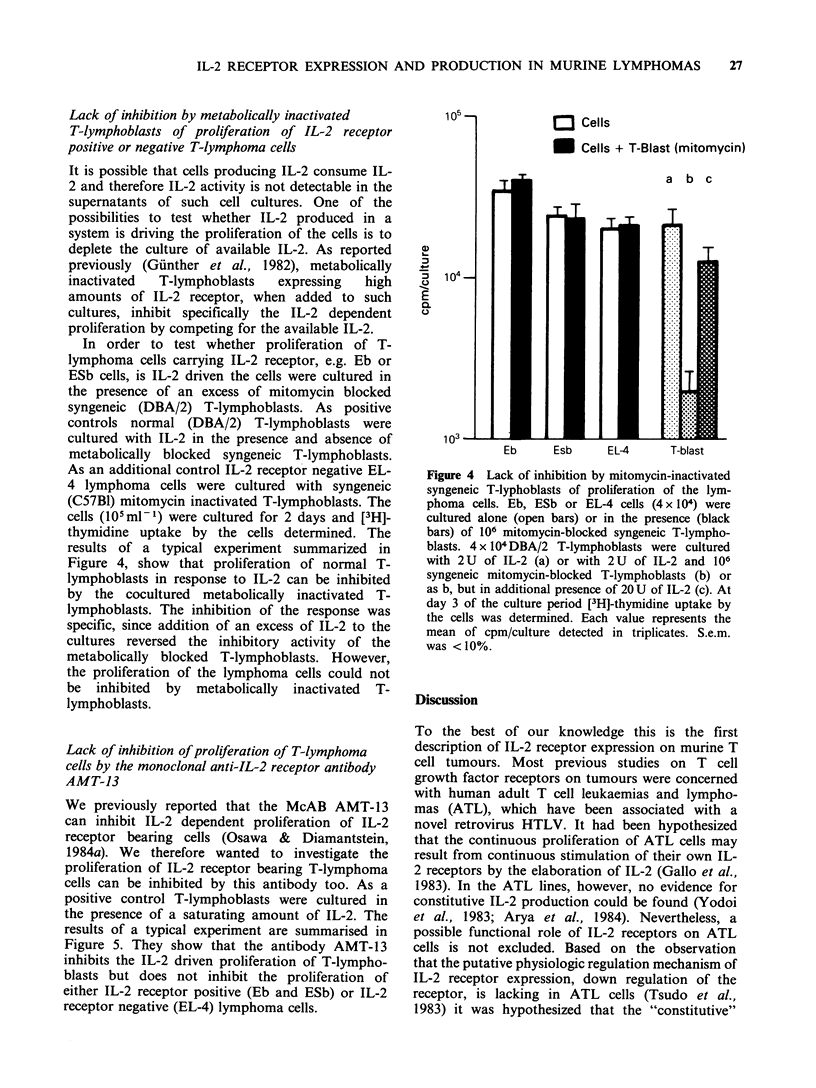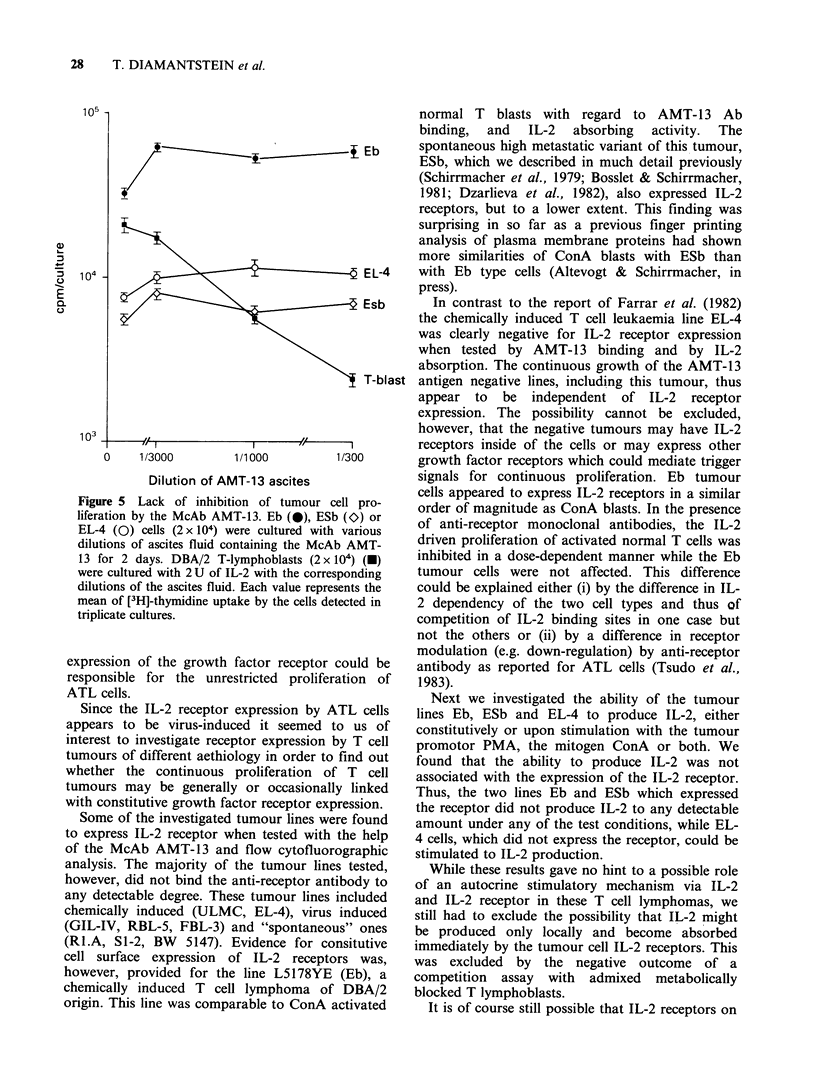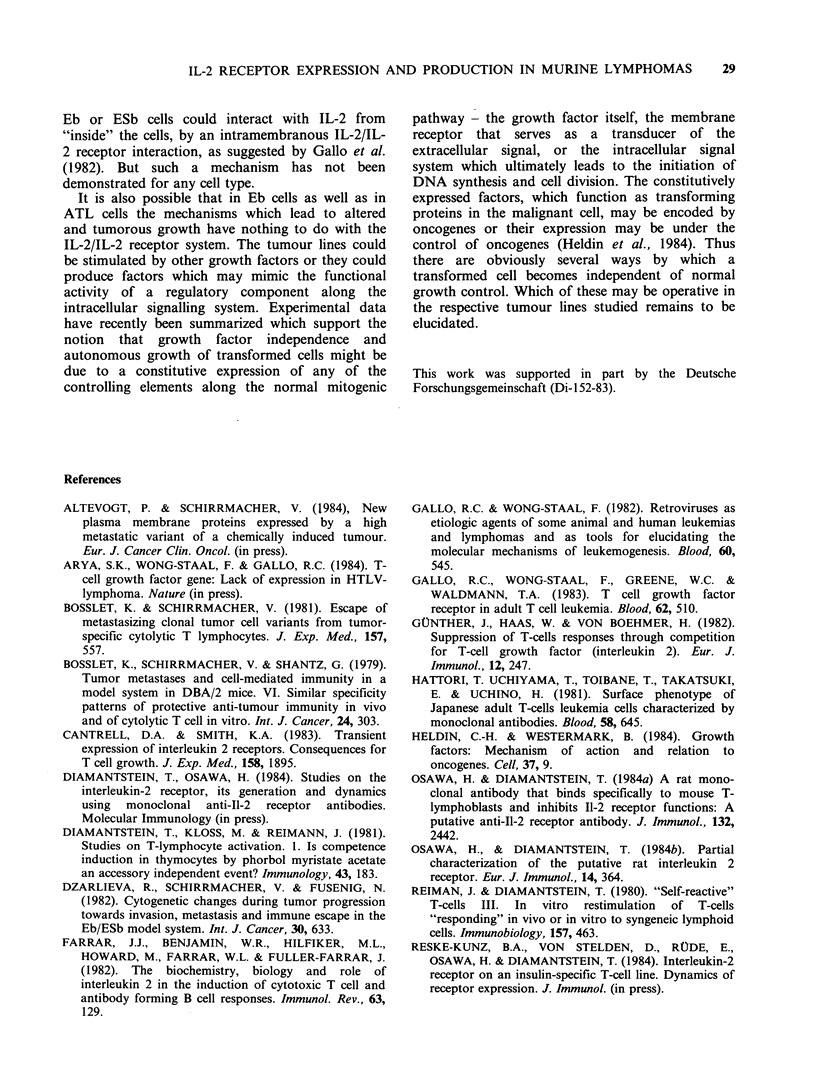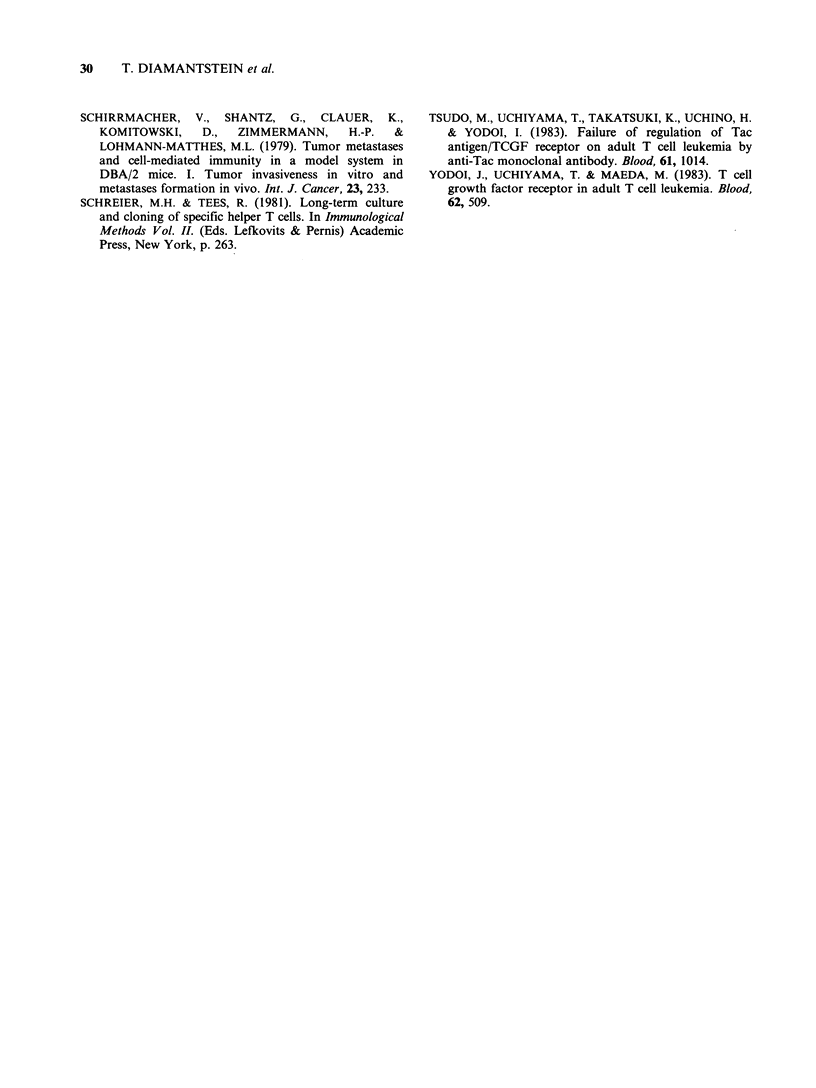# Studies on interleukin 2 receptor expression and IL-2 production by murine T cell lymphomas.

**DOI:** 10.1038/bjc.1985.4

**Published:** 1985-01

**Authors:** T. Diamantstein, H. Osawa, L. Graf, V. Schirrmacher

## Abstract

In order to study the possible role of the T-lymphocyte growth factor, Interleukin 2 (IL-2), and/or of the IL-2 receptor in the autonomous growth of leukaemic cells, 15 mouse leukaemic cell lines of various aetiology were analyzed for (i) IL-2 receptor expression and (ii) for the capacity to secrete IL-2. Several but not all of the cell lines tested were IL-2 receptor positive. The cells constitutively expressing IL-2 receptors at their surface could not be stimulated to secrete IL-2. Cell producing and secreting IL-2 did not express detectable amounts of IL-2 receptors at their surface. It has been demonstrated that proliferation of the leukaemic cells was independent of exogenous IL-2. The monoclonal anti-IL-2 receptor antibody AMT-13 inhibited IL-2 dependent proliferation of activated normal T-lymphocytes but failed to inhibit the growth of IL-2 receptor expressing leukaemic cells. The results argue against the autocrine stimulation hypothesis but do not exclude the possibility of involvement of functionally altered IL-2 receptors on autonomous cell growth.


*Br. J. Cancer* (1985), **51**, 23–30

### Studies on interleukin 2 receptor expression and IL-2 production by murine T cell lymphomas

T. Diamantstein[1], H. Osawa[1], L. Graf[2] & V. Schirrmacher[2]

[1]*Immunology Research Unit, Klinikum Steglitz, Freie Universität Berlin, 1000 Berlin 45,* [2]*Institut für Immunologie und Genetik, Deutsches Krebsforschungszentrum, 6900 Heidelberg, FRG*

**Summary** In order to study the possible role of the T-lymphocyte growth factor, Interleukin 2 (IL-2), and/or of the IL-2 receptor in the autonomous growth of leukaemic cells, 15 mouse leukaemic cell lines of various aetiology were analyzed for (i) IL-2 receptor expression and (ii) for the capacity to secrete IL-2. Several but not all of the cell lines tested were IL-2 receptor positive. The cells constitutively expressing IL-2 receptors at their surface could not be stimulated to secrete IL-2. Cell producing and secreting IL-2 did not express detectable amounts of IL-2 receptors at their surface. It has been demonstrated that proliferation of the leukaemic cells was independent of exogenous IL-2. The monoclonal anti-IL-2 receptor antibody AMT-13 inhibited IL-2 dependent proliferation of activated normal T-lymphocytes but failed to inhibit the growth of IL-2 receptor expressing leukaemic cells. The results argue against the autocrine stimulation hypothesis but do not exclude the possibility of involvement of functionally altered IL-2 receptors on autonomous cell growth.

For many, if not all cells, the initial trigger for proliferation appears to be the interaction of growth factors with the cell surface growth factor receptor. Activation of the growth factor receptor leads in turn to yet undefined cytoplasmic signalling systems.

Resting T lymphocytes are long living cells in the $G_0$ phase of the cell cycle. They only enter proliferative cycles under antigenic stimulation in the presence of a T cell growth factor, interleukin 2 (IL-2). Receptors for IL-2 are not detectable on the surface of resting T cells. Expression of IL-2 receptors is the consequence of interaction of antigen presenting cells with the antigen receptor. As shown recently, IL-2 receptor expression is a transient event and repeated restimulation by lectins (Cantrell & Smith, 1983; Dimantstain & Osawa, 1984) or the antigen (Reske-Kunz *et al.*, 1984) is required for continuous IL-2 receptor expression and consequently for long term cell growth.

In contrast to activated normal T-cells, adult T leukaemic cells (ATL cells) constitutively express IL-2 receptors (Hattori *et al.*, 1981; Yodoi *et al.*, 1983, Gallo *et al.*, 1983). It seemed plausible to us to ask whether the constitutive expression of the IL-2 receptor on leukaemic cells may be associated with the autonomous growth of the tumour cells. Recently we reported about the production and characterization of a monoclonal antibody (AMT-13) recognizing the IL-2 receptor present on the surface of activated murine T cells (Osawa & Diamantstein, 1984*a*, *b*). The aims of the present report were (i) to search for IL-2 receptor expression on murine T leukaemic cells of various aetiology and (ii) to search for arguments for or against the autocrine stimulation hypothesis (Gallo & Wong-Staal, 1982).

Correspondence: T. Diamantstein, Immunology Research Unit, Klinikum Steglitz, Freie Universität Berlin, D-1000 Berlin 45, Hindenburgdamm 27, FRG.
Received 14 August 1984.

### Materials and methods

### *Cell lines*

The aetiology and origin of most of the tumour lines used has been described (Bosslet *et al.*, 1979). The cell lines were grown in RPMI-1640 medium supplemented with 10% foetal calf serum and antibiotics as described (Bosslet *et al.*, 1979). Their characteristics relevant to the presented studies are indicated in Table I.

### *Preparation of cells*

T lymphoblasts were obtained by culturing $2 \times 10^6 ml^{-1}$ splenocytes with $3 \mu g ml^{-1}$ concanavalin A (ConA; Pharmacia Fine Chemicals, Uppsala, Sweden) in culture medium for 3 days. The cells were harvested and, to inactivate ConA, were treated with $20 mg ml^{-1}$ of α-methyl-mannoside as described previously (Reimann & Diamantstein, 1980). Metabolically blocked T-lymphoblasts were prepared by incubating T-lymphoblasts ($10^6 cells ml^{-1}$) with $50 \mu g ml^{-1}$ of mitomycin C at 37°C for 1 h as described previously (Reimann & Diamantstein, 1980).

© The Macmillan Press Ltd. 1985

*Cell cultures*

Cells ($10^5 ml^{-1}$) were cultured in a final volume of 0.2 ml in microtiter plates (Greiner, Nürtingen, FRG) in culture medium consisting of RPMI-1640 medium, supplemented with 5% FCS, $2 \times 10^{-3}$ M glutamine, streptomycin (50 $\mu g ml^{-1}$), penicillin (100 IU $ml^{-1}$) and $5 \times 10^{-5}$ M 2-mercaptoethanol. Cultures were incubated at 37°C in 5% $CO_2$ in air. Four hours before termination of the culture period, each well was pulsed with 10 $\mu l$ of [$^3$H]-thymidine ([$^3$H]-TdR, 0.1 $\mu$Ci, specific activity 2 Ci $mM^{-1}$, Radiochemical Centre, Amersham Buchler, Braunschweig, FRG). Uptake of [$^3$H]-TdR was determined as described (Diamantstein *et al.*, 1981). Each value represents the mean of counts per minute (cpm) detected in triplicate cultures. Standard error of mean was <10%. In several experiments, metabolically blocked T-lymphoblasts ($10^6$ cells $ml^{-1}$) or AMT-13 antibody in ascites form was added to the cultures. Appropriate concentrations of a control ascites fluid (Y3 ascites fluid) had no effect on the cell cultures.

*Preparation of semipurified IL-2*

IL-2 was prepared from the 48 h supernatants of rat spleen cells cultured with ConA by ammonium sulfate precipitation and Sephadex G-100 gel filtration exactly as described by Schreier and Tees (Schreier & Tees, 1981). This semipurified IL-2 was used throughout the experiments.

*Standard assay for IL-2 activity*

IL-2 activity was determined by IL-2 concentration-dependent proliferation of murine T lymphoblasts. The murine T lymphoblasts ($2 \times 10^4$ cells) were incubated in 0.2 ml of culture medium in the presence of serial twofold dilutions of a standard IL-2 preparation and the experimental sample. The cells were cultured for 72 h and [$^3$H]-TdR uptake by the cells determined. The dilution of the standard IL-2 preparation yielding 50% of the maximal [$^3$H]-thymidine uptake was defined as 1 U of IL-2.

*IL-2 absorption assay*

Cells ($5 \times 10^7$) were washed twice with PBS, and resuspended in 0.2 ml of IL-2 (100 U $ml^{-1}$). The suspensions were incubated for 1.5 h at 37°C. The cells were spun down, and the supernatants were assayed for remaining IL-2 activity as described above. The results were expressed as percentages of IL-2 absorbed by the respective cells.

*Fluorescence analysis*

For staining, $2 \times 10^6$ cells of various origin suspended in 0.1 ml of staining buffer (culture medium containing 0.1% $NaN_3$) were incubated for 30 min at 4°C with 0.1 ml of culture supernatant derived from the clone AMT-13. Controls were incubated with culture medium or supernatants derived from Y3 myeloma cells. The cells were washed with staining buffer and stained, if not otherwise stated, with an excess of FITC-conjugated $(Fab)_2$ fragment of sheep anti-rat Ig (E.Y., San Meteo, CA). The cells were washed, resuspended in 0.1 ml of staining buffer, and fixed with 0.7% paraformaldehyde. To detect cell surface-associated antigens recognized by AMT-13 McAb, the cells were subjected to flow cytofluorometry (FACS, Becton–Dickinson Type 400).

*Binding of $^{125}$I labelled second antibody to AMT-13 McAb binding cells*

This assay was performed in PBS containing 0.5% bovine serum albumin and 10 mM $NaN_3$. Cells ($0.5 \times 10^6$) were admixed with $5 \times 10^6$ sheep red blood cells (glutaraldehyde-stabilized, Sigma) in 1.5 ml medium in order to facilitate subsequent washings. The mixture was incubated for 30 min at 4°C. The cells were pelleted and resuspended in 100 $\mu l$ AMT-13 supernatant (dilution 1:3) or 100 $\mu l$ medium as a control. After an incubation period of 30 min at 4°C, the cells were washed with 1 ml of medium and resuspended in 100 $\mu l$ $^{125}$I-labelled sheep anti-rat Ig, $(Fab')_2$ fragment, diluted 1:10 (10 $\mu$CI $\mu g^{-1}$, 100 $\mu$CI $ml^{-1}$, Amersham Buchler, Braunschweig, FRG). Sixty minutes later, the cells were washed twice and the radioactivity associated with the cell pellet was determined in a Packard Gamma spectrometer. Each cell population was tested in triplicate.

*Attempts to induce IL-2 production*

Balb/c splenocytes or various lymphoma cell lines ($2 \times 10^6$ cells $ml^{-1}$) were incubated with 10 ng $ml^{-1}$ of phorbolmyristate acetate (PMA, Sigma München, FRG) or 3 $\mu g ml^{-1}$ of ConA or with a combination of PMA and ConA for 2 days (primary cultures). In order to defect IL-2 activity 0.02 ml of the respective culture supernatants containing 20 mg $ml^{-1}$ of $\alpha$-methylmannoside were added to 0.2 ml of T-lymphoblasts ($10^5$ cells $ml^{-1}$) and at day 3 of the culture period [$^3$H]-thymidine uptake by the cells detected (secondary cultures).

### Results

*Detection of IL-2 receptors on murine lymphoma cell lines*

In order to search for IL-2 receptor bearing lymphomas, cells of 13 different murine T-lymphoma

**Table I** Staining of murine lymphoma cell lines for IL-2 receptor expression using monoclonal antibody AMT-13 and a cytofluoro-graphic analysis

| *Tumour line* | | | *% positive cells*[b] | |
|---|---|---|---|---|
| *Designation* | *Type*[a] | *Strain* | *neg. control* | *AMT-13* |
| Rl. A | spont. thymoma | C58 | 1.3 (365) | 2.5 (265)[c] |
| El-4 | chem. lymph. (T) | C57B1/6 | 16.6 (263) | 14.8 (235) |
| SL-2 | spont. lymph. (T) | DBA/2 | 4.1 (245) | 14.2 (254)[c] |
| FBL-3 | virus lymph. (T) | C57B1/6 | 2.7 (198) | 6.9 (238)[c] |
| BW5147[TGR] | spont. lymph. (T) | AKR | 0.6 (308) | 2.6 (308)[c] |
| S-B10. RIII | spont. lymph. | 310. RIII | 10.9 (218) | 23.9 (241)[c] |
| GIL-IV | virus lymph. | C57B1/6 | 6.8 (209) | 7.2 (218) |
| LSTRA | virus lymph. | Balb/c | 11.1 (195) | 15.2 (191) |
| RBL-5 | virus lymph. (T) | C57B1/6 | 4.4 (151) | 4.2 (211) |
| ULMC | chem. lymph. | Balb/c | 6.9 (224) | 6.3 (210) |
| RLo-1 | rad. lymph. (T) | Balb/c | 9.0 (206) | 57.0 (261) |
| | | | 1.7 (174) | 95.2 (434)[c] |
| Eb | chem. lymph. (T) | DBA/2 | 14.8 (235) | 81.3 (601) |
| | | | 8.9 (220) | 92.9 (414)[c] |
| ESb | met. variant of Eb | DBA/2 | 21.4 (231) | 63.2 (326) |
| | | | 9.6 (215) | 48.0 (237)[c] |
| ESb-M | adh. variant of ESb | DBA/2 | 14.4 (182) | 52.0 (209)[c] |
| E24 | hybridoma of ESb | DBA/2 | 10.0 (223) | 37.8 (255)[c] |

[a]spont. = spontaneous; chem. = chemically induced; virus = virus induced; rad. = radiation induced; met. = metastatic; adh. = adherent; S-B10. RIII is a spontaneous lymphoma from our own colony of B10. RIII mice; the origin of the other tumour lines used is described elsewhere.

[b]The histograms consisted of 1000 channels, with channels 80–1000 counted as positive. Values in brackets are mean fluorescent intensity.

[c]Results of a test with rabbit anti rat Ig-FITC instead of $(Fab')_2$ – sheep anti rat Ig-FITC as second antibody. Negative control was second antibody alone.

lines were incubated with the monoclonal rat anti IL-2 receptor antibody AMT-13 (Osawa & Diamanstein, 1984*a*) and subsequently with FITC-conjugated anti-rat Ig. The results of the cytofluorometic analysis summarized in Table I show that some but not all lymphoma lines bind the McAb AMT-13 specifically.

Three out of the lymphoma cell lines were selected for a more detailed analysis: Eb-cells binding high level of AMT-13 antibody, Esb-cells binding a small but significant amount of AMT-13 antibody and EL-4 cells that failed to bind detectable amounts of AMT-13 antibody. The capacity of these cell lines to bind the antibody AMT-13 was compared to that of normal T-lymphoblasts by using a more sensitive indirect radioactive binding assay. Various numbers of the respective cells were incubated with a saturating amount of the McAb AMT-13 and with $^{125}I$-labelled $(Fab')_2$-fragments of anti-rat Ig as a second antibody. The results summarized in Figure 1 demonstrate that the Eb-cells and T-lymphoblast bind comparable amount of the McAb AMT-13. ESb cells bind the antibody AMT-13, only marginally as compared to Eb cells while El-4 cells were completely negative.

Binding of the McAb AMT-13 by activated T-cells has been shown to be paralleled by their capacity to absorb IL-2 activity (Osawa & Diamantstein, 1984*a*). As shown in Figure 2 cells such as T-lymphoblasts and Eb-cells carrying high numbers of binding sites for the McAb AMT-13 more effectively absorbed IL-2 activity than ESb-cells carrying small numbers of binding sites for this antibody. EL-4 cells and thymocytes did not absorb detectable amounts of IL-2 activity.

### *Attempts to induce IL-2 production by IL-2 receptor positive and IL-2 receptor negative T-lymphoma lines*

We asked whether IL-2 receptor expressing lymphoma cell lines are capable of producing IL-2. Eb-cells and ESb-cells that bind the McAb AMT-13 and EL-4 cells known to produce IL-2 but shown to lack binding sites for the McAb AMT-13 were cultured for 48 h with various doses of PMA

**Figure 1** Binding of [$^{125}$I]-labelled second antibody to cells preincubated with the McAb AMT-13. The indicated numbers of the different cells were incubated with hybridoma supernatant derived from the clone AMT-13 and subsequently with [$^{125}$I]-labelled $F(ab')_2$ fragment of sheep anti rat Ig. In triplicates, the radioactivity associated with the cells was determined. S.e.m. was less than 5%.

**Figure 2** Absorption of IL-2 activity by tumour cell lines. Cells ($5 \times 10^7$) were incubated with 20 U IL-2 and the remaining IL-2 activity in the respective supernatants was tested as described in *Materials and methods*. The results were expressed as percentages of IL-2 absorbed. Four independent experiments gave similar results.

(in dose range of 1–100 ng PMA per $2 \times 10^6$ cells ml$^{-1}$) or with various doses of ConA (1–10 $\mu$g ConA per $2 \times 10^6$ cells ml$^{-1}$) or a combination of PMA and ConA (primary cultures). Cell free supernatants derived from primary cultures containing $\alpha$-methylmannoside (20 mg ml$^{-1}$) were then tested for IL-2 activity. The results of a typical experiment using optimum stimulatory doses of PMA and ConA are summerized in Figure 3. Stimulation of EL-4 cells for IL-2 production was achieved by incubating the cells with 10 ng PMA but not with ConA. Combination of 10 ng PMA and 3 $\mu$g ConA gave optimum stimulation. Neither of the PMA or ConA doses added separately or in combination to Eb or ESb cells induced detectable amounts of IL-2.

**Figure 3** Lack of IL-2 production by Eb and ESb tumour cell lines. Two million cells ml$^{-1}$ were cultured as indicated with medium (—), PMA (10 ng), ConA (3 $\mu$g) or combination of 10 ng PMA and 3 $\mu$g ConA for 2 day (primary cultures). Supernatants containing 20 mg/ml$^{-1}$ of $\alpha$-methyl mannoside were then tested in secondary cultures for IL-2 activity using $2 \times 10^4$ Balb/c T-lymphoblasts as detector cells. Each value represents the mean of cpm/culture detected in triplicates. S.e.m. was $< 10\%$. Three separate experiments gave similar results.

*Lack of inhibition by metabolically inactivated T-lymphoblasts of proliferation of IL-2 receptor positive or negative T-lymphoma cells*

It is possible that cells producing IL-2 consume IL-2 and therefore IL-2 activity is not detectable in the supernatants of such cell cultures. One of the possibilities to test whether IL-2 produced in a system is driving the proliferation of the cells is to deplete the culture of available IL-2. As reported previously (Günther *et al.,* 1982), metabolically inactivated T-lymphoblasts expressing high amounts of IL-2 receptor, when added to such cultures, inhibit specifically the IL-2 dependent proliferation by competing for the available IL-2.

In order to test whether proliferation of T-lymphoma cells carrying IL-2 receptor, e.g. Eb or ESb cells, is IL-2 driven the cells were cultured in the presence of an excess of mitomycin blocked syngeneic (DBA/2) T-lymphoblasts. As positive controls normal (DBA/2) T-lymphoblasts were cultured with IL-2 in the presence and absence of metabolically blocked syngeneic T-lymphoblasts. As an additional control IL-2 receptor negative EL-4 lymphoma cells were cultured with syngeneic (C57Bl) mitomycin inactivated T-lymphoblasts. The cells ($10^5\,ml^{-1}$) were cultured for 2 days and [$^3$H]-thymidine uptake by the cells determined. The results of a typical experiment summarized in Figure 4, show that proliferation of normal T-lymphoblasts in response to IL-2 can be inhibited by the cocultured metabolically inactivated T-lymphoblasts. The inhibition of the response was specific, since addition of an excess of IL-2 to the cultures reversed the inhibitory activity of the metabolically blocked T-lymphoblasts. However, the proliferation of the lymphoma cells could not be inhibited by metabolically inactivated T-lymphoblasts.

*Lack of inhibition of proliferation of T-lymphoma cells by the monoclonal anti-IL-2 receptor antibody AMT-13*

We previously reported that the McAB AMT-13 can inhibit IL-2 dependent proliferation of IL-2 receptor bearing cells (Osawa & Diamantstein, 1984*a*). We therefore wanted to investigate the proliferation of IL-2 receptor bearing T-lymphoma cells can be inhibited by this antibody too. As a positive control T-lymphoblasts were cultured in the presence of a saturating amount of IL-2. The results of a typical experiment are summarised in Figure 5. They show that the antibody AMT-13 inhibits the IL-2 driven proliferation of T-lymphoblasts but does not inhibit the proliferation of either IL-2 receptor positive (Eb and ESb) or IL-2 receptor negative (EL-4) lymphoma cells.

**Figure 4** Lack of inhibition by mitomycin-inactivated syngeneic T-lyphoblasts of proliferation of the lymphoma cells. Eb, ESb or EL-4 cells ($4\times10^4$) were cultured alone (open bars) or in the presence (black bars) of $10^6$ mitomycin-blocked syngeneic T-lymphoblasts. $4\times10^4$ DBA/2 T-lymphoblasts were cultured with 2 U of IL-2 (a) or with 2 U of IL-2 and $10^6$ syngeneic mitomycin-blocked T-lymphoblasts (b) or as b, but in additional presence of 20 U of IL-2 (c). At day 3 of the culture period [$^3$H]-thymidine uptake by the cells was determined. Each value represents the mean of cpm/culture detected in triplicates. S.e.m. was $<10\%$.

### Discussion

To the best of our knowledge this is the first description of IL-2 receptor expression on murine T cell tumours. Most previous studies on T cell growth factor receptors on tumours were concerned with human adult T cell leukaemias and lymphomas (ATL), which have been associated with a novel retrovirus HTLV. It had been hypothesized that the continuous proliferation of ATL cells may result from continuous stimulation of their own IL-2 receptors by the elaboration of IL-2 (Gallo *et al.,* 1983). In the ATL lines, however, no evidence for constitutive IL-2 production could be found (Yodoi *et al.,* 1983; Arya *et al.,* 1984). Nevertheless, a possible functional role of IL-2 receptors on ATL cells is not excluded. Based on the observation that the putative physiologic regulation mechanism of IL-2 receptor expression, down regulation of the receptor, is lacking in ATL cells (Tsudo *et al.,* 1983) it was hypothesized that the "constitutive"

**Figure 5** Lack of inhibition of tumour cell proliferation by the McAb AMT-13. Eb (●), ESb (◇) or EL-4 (○) cells ($2\times10^4$) were cultured with various dilutions of ascites fluid containing the McAb AMT-13 for 2 days. DBA/2 T-lymphoblasts ($2\times10^4$) (■) were cultured with 2 U of IL-2 with the corresponding dilutions of the ascites fluid. Each value represents the mean of [$^3$H]-thymidine uptake by the cells detected in triplicate cultures.

expression of the growth factor receptor could be responsible for the unrestricted proliferation of ATL cells.

Since the IL-2 receptor expression by ATL cells appears to be virus-induced it seemed to us of interest to investigate receptor expression by T cell tumours of different aethiology in order to find out whether the continuous proliferation of T cell tumours may be generally or occasionally linked with constitutive growth factor receptor expression.

Some of the investigated tumour lines were found to express IL-2 receptor when tested with the help of the McAb AMT-13 and flow cytofluorographic analysis. The majority of the tumour lines tested, however, did not bind the anti-receptor antibody to any detectable degree. These tumour lines included chemically induced (ULMC, EL-4), virus induced (GIL-IV, RBL-5, FBL-3) and "spontaneous" ones (R1.A, S1-2, BW 5147). Evidence for consitutive cell surface expression of IL-2 receptors was, however, provided for the line L5178YE (Eb), a chemically induced T cell lymphoma of DBA/2 origin. This line was comparable to ConA activated normal T blasts with regard to AMT-13 Ab binding, and IL-2 absorbing activity. The spontaneous high metastatic variant of this tumour, ESb, which we described in much detail previously (Schirrmacher *et al.*, 1979; Bosslet & Schirrmacher, 1981; Dzarlieva *et al.*, 1982), also expressed IL-2 receptors, but to a lower extent. This finding was surprising in so far as a previous finger printing analysis of plasma membrane proteins had shown more similarities of ConA blasts with ESb than with Eb type cells (Altevogt & Schirrmacher, in press).

In contrast to the report of Farrar *et al.* (1982) the chemically induced T cell leukaemia line EL-4 was clearly negative for IL-2 receptor expression when tested by AMT-13 binding and by IL-2 absorption. The continuous growth of the AMT-13 antigen negative lines, including this tumour, thus appear to be independent of IL-2 receptor expression. The possibility cannot be excluded, however, that the negative tumours may have IL-2 receptors inside of the cells or may express other growth factor receptors which could mediate trigger signals for continuous proliferation. Eb tumour cells appeared to express IL-2 receptors in a similar order of magnitude as ConA blasts. In the presence of anti-receptor monoclonal antibodies, the IL-2 driven proliferation of activated normal T cells was inhibited in a dose-dependent manner while the Eb tumour cells were not affected. This difference could be explained either (i) by the difference in IL-2 dependency of the two cell types and thus of competition of IL-2 binding sites in one case but not the others or (ii) by a difference in receptor modulation (e.g. down-regulation) by anti-receptor antibody as reported for ATL cells (Tsudo *et al.*, 1983).

Next we investigated the ability of the tumour lines Eb, ESb and EL-4 to produce IL-2, either constitutively or upon stimulation with the tumour promotor PMA, the mitogen ConA or both. We found that the ability to produce IL-2 was not associated with the expression of the IL-2 receptor. Thus, the two lines Eb and ESb which expressed the receptor did not produce IL-2 to any detectable amount under any of the test conditions, while EL-4 cells, which did not express the receptor, could be stimulated to IL-2 production.

While these results gave no hint to a possible role of an autocrine stimulatory mechanism via IL-2 and IL-2 receptor in these T cell lymphomas, we still had to exclude the possibility that IL-2 might be produced only locally and become absorbed immediately by the tumour cell IL-2 receptors. This was excluded by the negative outcome of a competition assay with admixed metabolically blocked T lymphoblasts.

It is of course still possible that IL-2 receptors on

Eb or ESb cells could interact with IL-2 from "inside" the cells, by an intramembranous IL-2/IL-2 receptor interaction, as suggested by Gallo *et al.* (1982). But such a mechanism has not been demonstrated for any cell type.

It is also possible that in Eb cells as well as in ATL cells the mechanisms which lead to altered and tumorous growth have nothing to do with the IL-2/IL-2 receptor system. The tumour lines could be stimulated by other growth factors or they could produce factors which may mimic the functional activity of a regulatory component along the intracellular signalling system. Experimental data have recently been summarized which support the notion that growth factor independence and autonomous growth of transformed cells might be due to a constitutive expression of any of the controlling elements along the normal mitogenic pathway – the growth factor itself, the membrane receptor that serves as a transducer of the extracellular signal, or the intracellular signal system which ultimately leads to the initiation of DNA synthesis and cell division. The constitutively expressed factors, which function as transforming proteins in the malignant cell, may be encoded by oncogenes or their expression may be under the control of oncogenes (Heldin *et al.*, 1984). Thus there are obviously several ways by which a transformed cell becomes independent of normal growth control. Which of these may be operative in the respective tumour lines studied remains to be elucidated.

This work was supported in part by the Deutsche Forschungsgemeinschaft (Di-152-83).

### References

ALTEVOGT, P. & SCHIRRMACHER, V. (1984), New plasma membrane proteins expressed by a high metastatic variant of a chemically induced tumour. *Eur. J. Cancer Clin. Oncol.* (in press).

ARYA, S.K., WONG-STAAL, F. & GALLO, R.C. (1984). T-cell growth factor gene: Lack of expression in HTLV-lymphoma. *Nature* (in press).

BOSSLET, K. & SCHIRRMACHER, V. (1981). Escape of metastasizing clonal tumor cell variants from tumor-specific cytolytic T lymphocytes. *J. Exp. Med.*, **157**, 557.

BOSSLET, K., SCHIRRMACHER, V. & SHANTZ, G. (1979). Tumor metastases and cell-mediated immunity in a model system in DBA/2 mice. VI. Similar specificity patterns of protective anti-tumour immunity in vivo and of cytolytic T cell in vitro. *Int. J. Cancer*, **24**, 303.

CANTRELL, D.A. & SMITH, K.A. (1983). Transient expression of interleukin 2 receptors. Consequences for T cell growth. *J. Exp. Med.*, **158**, 1895.

DIAMANTSTEIN, T., OSAWA, H. (1984). Studies on the interleukin-2 receptor, its generation and dynamics using monoclonal anti-Il-2 receptor antibodies. Molecular Immunology (in press).

DIAMANTSTEIN, T., KLOSS, M. & REIMANN, J. (1981). Studies on T-lymphocyte activation. 1. Is competence induction in thymocytes by phorbol myristate acetate an accessory independent event? *Immunology*, **43**, 183.

DZARLIEVA, R., SCHIRRMACHER, V. & FUSENIG, N. (1982). Cytogenetic changes during tumor progression towards invasion, metastasis and immune escape in the Eb/ESb model system. *Int. J. Cancer*, **30**, 633.

FARRAR, J.J., BENJAMIN, W.R., HILFIKER, M.L., HOWARD, M., FARRAR, W.L. & FULLER-FARRAR, J. (1982). The biochemistry, biology and role of interleukin 2 in the induction of cytotoxic T cell and antibody forming B cell responses. *Immunol. Rev.*, **63**, 129.

GALLO, R.C. & WONG-STAAL, F. (1982). Retroviruses as etiologic agents of some animal and human leukemias and lymphomas and as tools for elucidating the molecular mechanisms of leukemogenesis. *Blood*, **60**, 545.

GALLO, R.C., WONG-STAAL, F., GREENE, W.C. & WALDMANN, T.A. (1983). T cell growth factor receptor in adult T cell leukemia. *Blood*, **62**, 510.

GÜNTHER, J., HAAS, W. & VON BOEHMER, H. (1982). Suppression of T-cells responses through competition for T-cell growth factor (interleukin 2). *Eur. J. Immunol.*, **12**, 247.

HATTORI, T. UCHIYAMA, T., TOIBANE, T., TAKATSUKI, E. & UCHINO, H. (1981). Surface phenotype of Japanese adult T-cells leukemia cells characterized by monoclonal antibodies. *Blood*, **58**, 645.

HELDIN, C.-H. & WESTERMARK, B. (1984). Growth factors: Mechanism of action and relation to oncogenes. *Cell*, **37**, 9.

OSAWA, H. & DIAMANTSTEIN, T. (1984*a*) A rat monoclonal antibody that binds specifically to mouse T-lymphoblasts and inhibits Il-2 receptor functions: A putative anti-Il-2 receptor antibody. *J. Immunol.*, **132**, 2442.

OSAWA, H., & DIAMANTSTEIN, T. (1984*b*). Partial characterization of the putative rat interleukin 2 receptor. *Eur. J. Immunol.*, **14**, 364.

REIMAN, J. & DIAMANTSTEIN, T. (1980). "Self-reactive" T-cells III. In vitro restimulation of T-cells "responding" in vivo or in vitro to syngeneic lymphoid cells. *Immunobiology*, **157**, 463.

RESKE-KUNZ, B.A., VON STELDEN, D., RÜDE, E., OSAWA, H. & DIAMANTSTEIN, T. (1984). Interleukin-2 receptor on an insulin-specific T-cell line. Dynamics of receptor expression. *J. Immunol.* (in press).

SCHIRRMACHER, V., SHANTZ, G., CLAUER, K., KOMITOWSKI, D., ZIMMERMANN, H.-P. & LOHMANN-MATTHES, M.L. (1979). Tumor metastases and cell-mediated immunity in a model system in DBA/2 mice. I. Tumor invasiveness in vitro and metastases formation in vivo. *Int. J. Cancer,* **23,** 233.

SCHREIER, M.H. & TEES, R. (1981). Long-term culture and cloning of specific helper T cells. In *Immunological Methods Vol. II.* (Eds. Lefkovits & Pernis) Academic Press, New York, p. 263.

TSUDO, M., UCHIYAMA, T., TAKATSUKI, K., UCHINO, H. & YODOI, I. (1983). Failure of regulation of Tac antigen/TCGF receptor on adult T cell leukemia by anti-Tac monoclonal antibody. *Blood,* **61,** 1014.

YODOI, J., UCHIYAMA, T. & MAEDA, M. (1983). T cell growth factor receptor in adult T cell leukemia. *Blood,* **62,** 509.